# Nephrostomy catheter entering the right renal vein during an exchange procedure: A case report and literature review

**DOI:** 10.1002/iju5.12276

**Published:** 2021-03-11

**Authors:** Jun Akatsuka, Yasutomo Suzuki, Yuki Endo, Masato Yanagi, Ichiro Matsuzawa, Tsutomu Hamasaki, Go Kimura, Yukihiro Kondo

**Affiliations:** ^1^ Department of Urology Nippon Medical School Tokyo Japan

**Keywords:** complications, hemorrhage, nephrostomy

## Abstract

**Introduction:**

We encountered an extremely rare case of a nephrostomy catheter entering the right renal vein during an exchange procedure.

**Case presentation:**

An 80‐year‐old man underwent radical cystectomy. Urinary diversion was achieved through right percutaneous nephrostomy. After the 15^th^ nephrostomy catheter exchange, the patient bled heavily from the catheter. We clamped the catheter immediately, and the patient became hemodynamically stable. Emergency angiography showed the nephrostomy catheter entering the renal vein from outside the renal pelvis. Under fluoroscopy, we pulled the catheter until its tip was located in the previous penetration site of the renal pelvic wall and inserted the catheter over the guidewire into the renal pelvis.

**Conclusions:**

Herein, we report an extremely rare case of a nephrostomy catheter inserted into the right renal vein during an exchange procedure. Inserting a nephrostomy catheter in the appropriate position and performing exchange under imaging guidance techniques could help clinicians avoid severe complications.

**Abbreviations & Acronyms**
CT: computed tomography
IVC: inferior vena cava
PCNL: percutaneous nephrolithotomy

**Keynote message**

We have reported an extremely rare case of a nephrostomy catheter entering the renal vein during an exchange procedure. Inserting a nephrostomy catheter in the appropriate position and performing exchange under imaging guidance techniques could avoid severe complications.

## Introduction

Percutaneous nephrostomy is a highly effective interventional procedure most commonly performed to relieve hydronephrosis. There are various possible complications of this procedure, including hemorrhage, sepsis, and pneumothorax.^1^ One of the most severe complications is penetration into the vascular system by the catheter, of which there are few reports in the literature.^2^, ^3^, ^4^, ^5^, ^6^, ^7^, ^8^, ^9^, ^10^ Most reported complications occur following PCNL.^2^, ^3^, ^4^, ^5^, ^6^, ^7^ There are few reports of such a complication during catheter exchange.^8^, ^9^ We report an extremely rare case of a nephrostomy catheter entering the vascular system during an exchange procedure.

## Case presentation

**Fig. 1:**
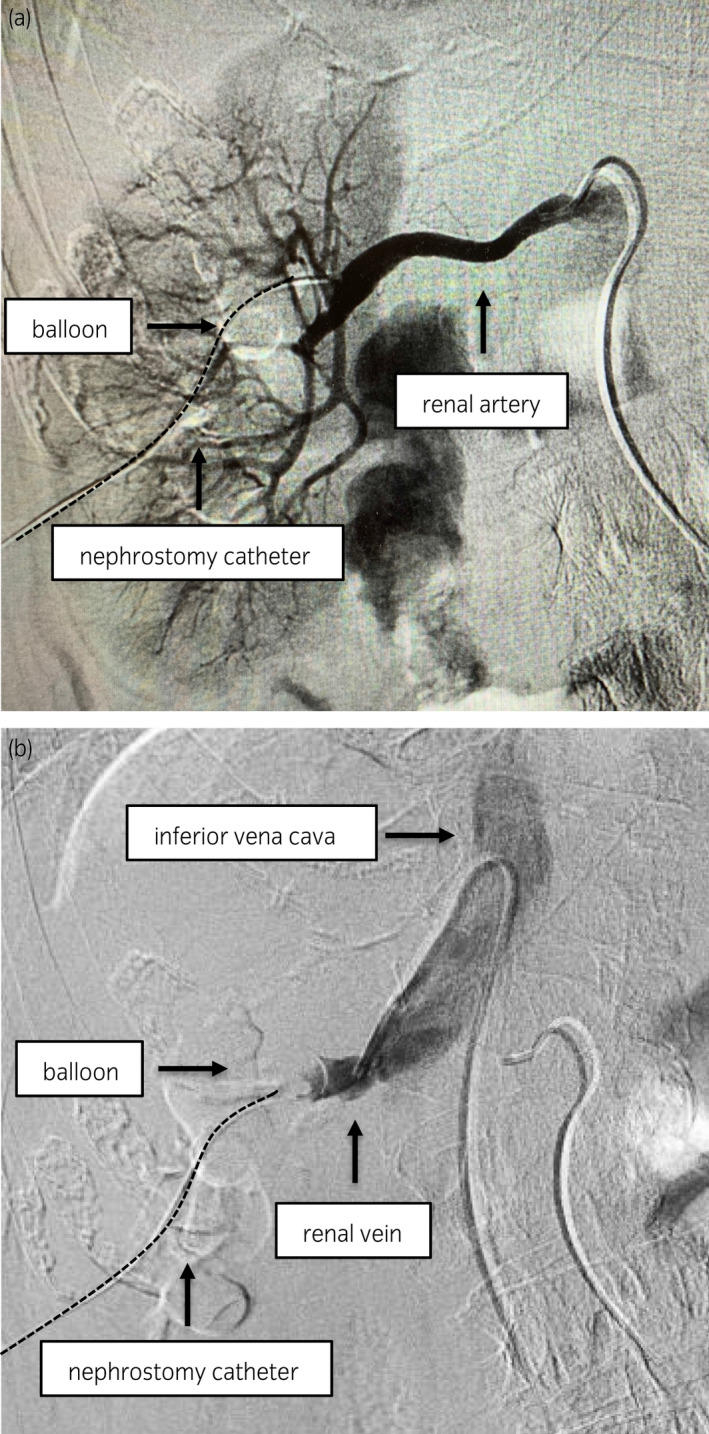
Renal angiography following insertion of the catheter into the renal vein. (a) Renal arteriography. Renal arteriography demonstrated no arterial bleeding. (b) Renal venography. On the emergency renal venography, the nephrostomy catheter’s dilated balloon is observed in the renal vein.

**Fig. 2:**
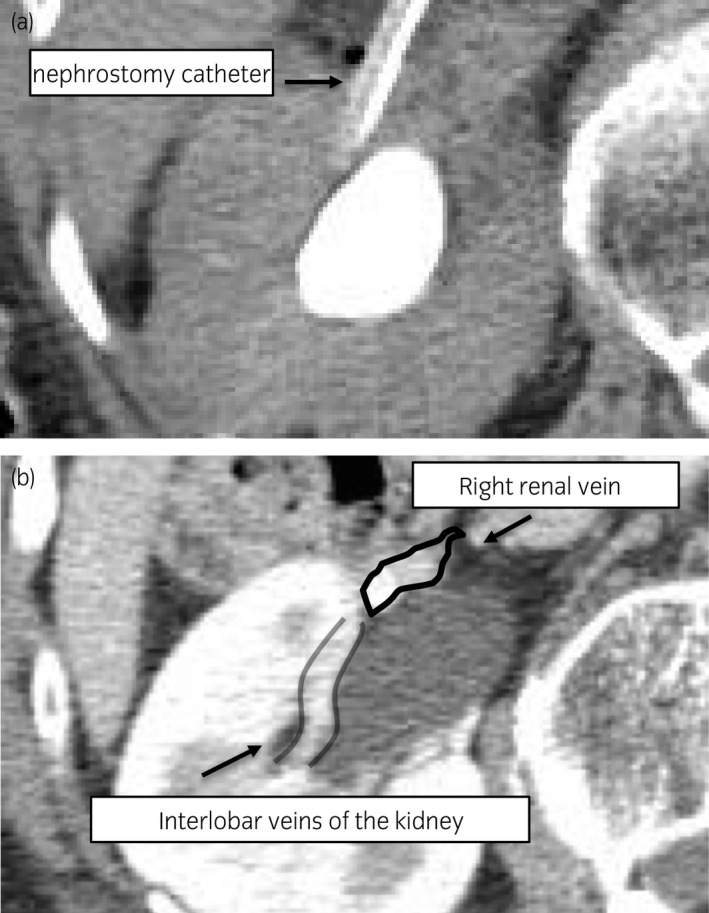
Abdominal CT scan. (a) Abdominal CT scan shows the nephrostomy catheter straying into the renal vein outside the renal pelvis. The contrast agent used in the angiography is seen only in the renal pelvis, and no extravasation of urine or bleeding from the renal pelvis is observed. (b) Abdominal CT scan before this event shows the interlobar veins of the kidney at the right ventral renal pelvis.

**Fig. 3:**
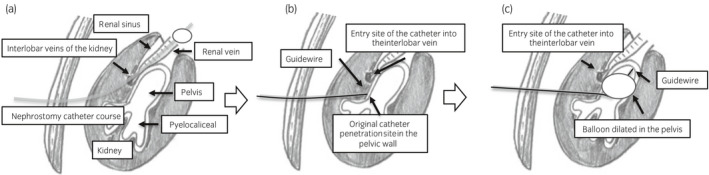
Anticipated route of the catheter into the renal vein and the recovery procedure for this event in the present case. (a) Schematic diagram of the anticipated route of the nephrostomy catheter into the renal vein: we speculated that the catheter might have entered the renal vein via the interlobar vein, through the outside of the renal pelvis. Schematic diagram of the recovery procedure for this event. (b) The catheter was drawn out along a guidewire and was stopped at the original site at which the pelvic wall had been penetrated. (c) We advanced the guidewire into the renal pelvic space through the previous tract and inserted the catheter over the guidewire into the renal pelvis. Finally, the catheter was fixed at the puncture site in the renal pelvis.

An 80‐year‐old man had undergone with a nephrostomy for post‐renal renal failure due to a bladder tumor around the right ureteral orifice with a contralateral atrophic kidney. Thereafter, the patient underwent radical cystectomy because of a diagnosis of invasive bladder cancer, and nephrostomy was chosen for urinary diversion owing to the patient’s preference. A year later, in the outpatient clinic, we performed routine exchange of his 18‐Fr silicon catheter, but the urinary tract was resistant to the exchange. We attempted insertion again by switching from the 18‐Fr to a 14‐Fr silicon catheter, while paying attention to the catheter’s depth of insertion and its resistance to insertion. All of these procedures were performed without medical equipment such as guidewire. After these procedures, we noticed a discharge that contained blood. We instructed the patient to remain in bed and, 10 min after the procedure, we noticed continuous bleeding from the catheter. The patient subsequently went into hypovolemic shock. We immediately clamped the catheter and started rapid intravenous saline infusion. After confirming the patient's hemodynamic stability, emergency renal angiography was performed. We located the inflated balloon of the nephrostomy catheter inside the renal vein (Fig. 1a,b). On conducting abdominal CT after angiography, it was evident that the nephrostomy catheter had strayed into the renal vein from the right ventral renal pelvis, with the contrast agent used in the angiography visible only in the renal pelvis. No extravasation of urine or bleeding from the renal pelvis was observed (Fig. 2a). Compared with the previous CT, the nephrostomy catheter had dislodged into the interlobar veins of the kidney (Fig. 2b). We performed a recovery procedure under general anesthesia in the operating room. Under fluoroscopy, the renal pelvis was filled with the contrast agent, which had been used during the angiography. We discovered that the nephrostomy catheter had entered the interlobar vein from the outside the renal pelvis (Fig. 3a). We pulled the catheter carefully using a guidewire until the catheter tip was within the penetration site of the renal pelvic wall (Fig. 3b). We advanced the guidewire into the renal pelvic space through the previous tract, inserted the catheter over the guidewire into the renal pelvis, and fixed it at the position of the puncture site in the renal pelvis (Fig. 3c). The inflated balloon compressed the damaged interlobar vein from the side of the renal pelvis. After releasing the clamp on the nephrostomy catheter, the flow of transparent yellow urine was confirmed. Fifteen days after the event, we replaced the nephrostomy catheter with a new one into a lower calyx through the renal papilla.

## Discussion

Ours is the 14^th^ reported case of a misplaced nephrostomy catheter in the vascular system and the third to have occurred during an exchange procedure (Table 1).^2^, ^3^, ^4^, ^5^, ^6^, ^7^, ^8^, ^9^, ^10^ In 10 of 14 cases, the misplacement occurred after PCNL. Mazzucchi *et al*. reported two cases of intravenous misplacement of the nephrostomy catheter after PCNL as uncommon complications and provided details of their management.^2^ Chen *et al*. reported three cases, and the incidence of intravenous misplacement of the nephrostomy catheter after PCNL was 0.5% (2/4148 cases).^3^ However, there are a few reports of this complication occurring during catheter exchange. Dias‐Filho *et al*. reported a case of inadvertent renal vein penetration during nephrostomy catheter exchange, with right atrial migration of the catheter.^8^ Kotb *et al*. reported a case of percutaneous silicon catheter insertion into the IVC. They concluded that a nephrostomy catheter should be exchanged under ultrasound or fluoroscopic guidance and argued against inserting any catheter into the kidney in a blind manner, even into a mature tract.^9^ We misplaced the nephrostomy catheter, which entered the renal vein, with a blinded technique during the 15^th^ exchange.

**Table 1:** Reported cases of nephrostomy catheter misplacement

No	Author	Year	Age	Sex	Side	Location	Occasion	Operation type
1	Dias‐Filho *et al*.^8^	2005	63	F	L	Renal vein/IVC/right atrium	Exchanging	Noninvasive procedure
2	Shaw *et al*.^4^	2005	54	M	R	Renal vein	PCNL	Exploratory
3	Mazzucchi *et al*.^2^	2009	52	M	L	Renal vein	PCNL	Noninvasive procedure
4	Mazzucchi *et al*.^2^	2009	35	F	L	Renal vein/IVC	PCNL	Noninvasive procedure
5	Li *et al*.^5^	2013	32	F	L	Renal vein/IVC	PCNL	Noninvasive procedure
6	Kotb *et al*.^9^	2013	50	M	L	Renal vein/IVC	Exchanging	Open pyelotomy
7	Wang *et al*.^6^	2013	66	F	L	Renal vein	PCNL	Noninvasive procedure
8	Chen *et al*.^3^	2014	42	M	L	Renal vein/IVC	PCNL	Noninvasive procedure
9	Chen *et al*.^3^	2014	38	F	L	Renal vein/IVC	PCNL	Noninvasive procedure
10	Chen *et al*.^3^	2014	48	M	L	Renal vein	PCNL	Noninvasive procedure
11	Al Zahrani *et al*.^10^	2016	76	F	R	Renal vein/IVC	Insertion	Noninvasive procedure
12	Fu *et al*.^7^	2017	68	M	R	Renal vein	PCNL	Exploratory laparotomy
13	Fu *et al*.^7^	2017	28	M	L	Renal vein/IVC	PCNL	Exploratory laparotomy
14	Our case	2021	80	M	R	Renal vein	Exchanging	Noninvasive procedure

F, female; M, male; L, left; R, right.

Several reports have described using a relatively noninvasive procedure to prevent further complications, with a suggested management as follows: once intravenous misplacement is detected, the catheter should be clamped immediately, and the clamped catheter should be drawn back and repositioned in the collecting system under image guidance.^3^ Zahrani *et al*. reported a case of obstructive uropathy in a solitary kidney where placement of a nephrostomy catheter was complicated by its insertion into the IVC. They performed controlled removal of the misplaced nephrostomy catheter utilizing the percutaneous intravenous balloon tamponade technique.^10^ However, Fu *et al*. performed open surgery under general anesthesia, mainly to remove residual calculi and to prepare for any possible adverse events.^7^ In our case, a CT scan showed no bleeding into the renal pelvis or the retroperitoneal space. Therefore, the nephrostomy catheter was drawn outward, and the tip was adjusted to a position in the renal pelvis adjacent to the site of injury, using fluoroscopy under general anesthesia. As a result, we avoided further severe complications.

We considered the following issues in our management. First, the initial insertion position was inadequate. This insertion was made not through the renal papilla but directly into the renal pelvis. This procedure is risky and not recommended due to the possibility of vessel damage between the renal parenchyma and the renal pelvis. Inserting the catheter in the appropriate site not only provides good drainage but also reduces complications during placement and the exchange procedure. Second, even when the soft silicone catheter is replaced, exchanging the catheter using the blinded technique can lead to its insertion of the catheter into the incorrect area. Due to the long‐term nephrostomy management, the renal tissues in contact with the catheter may have become fragile.

We have reported an extremely rare case regarding a misplaced nephrostomy catheter extending into the renal vein during an exchange procedure. Even during an exchange procedure, intravenous misplacement of the nephrostomy catheter can occur. To avoid this severe complication, the catheter must be inserted at the appropriate position and exchanged under the guidance of imaging techniques such as ultrasound or fluoroscopy.

## Conflict of interest

The authors declare no conflict of interest.
